# Research on the long tail mechanism of digital finance alleviating the relative poverty of rural households

**DOI:** 10.1371/journal.pone.0284988

**Published:** 2023-04-27

**Authors:** Shengfeng Xie, Caimin Jin, Ting Song, Chengxiao Feng

**Affiliations:** 1 School of Business, Hubei University, Wuhan, Hubei Province, P.R. China; 2 School of Computer Science and Information Engineering, Hubei University, Wuhan, Hubei Province, P.R. China; 3 School of Foreign Languages, Hubei University, Wuhan, Hubei Province, P.R. China; Szechenyi Istvan University: Szechenyi Istvan Egyetem, HUNGARY

## Abstract

Digital finance provides a long-tail mechanism for alleviating relative poverty caused by unequal opportunities and rights. According to the inference of an improved Cobb-Douglas production function and Ramsey-Cass-Koopmans two-stage household consumption model, the long-tail mechanism for digital finance to alleviate the relative poverty of farmers includes productive investment mechanism, credit mechanism, financial asset allocation and entrepreneurial mechanism. An empirical analysis of 11,519 rural households across China based on CHFS2019 data shows that digital finance can significantly and steadily alleviate relative poverty by improving credit availability and promoting household entrepreneurship, while its effect on increasing productive investment opportunities and optimizing financial asset allocation is less certain. Therefore, it is necessary to continue to improve the "blood making" long tail mechanism of digital finance for farmers’ credit and innovation and entrepreneurship, and at the same time guide the digital finance to empower the development of rural industries to increase farmers’ productive investment opportunities, cultivate endogenous growth momentum, and improve the wealth allocation function of rural digital financial market.

## 1 Introduction

In the post-epidemic era with the transition from old to new driving forces, digital finance dominated by digitalization and "Internet +" has gradually become the mainstream under the catalysis of the epidemic and has become the representative of the digital platform economy. Compared with the traditional financial poverty reduction mechanism, digital finance changes the way of financial services, reduces the risk of information asymmetry and the finance cost, relying on mobile internet, technology platform, big data, cloud computing, artificial intelligence and block chain technology. For the relatively poor in rural, digital finance not only has low entry barriers and transaction costs, but also is more precise and targeted. It reflects the sharing and fair long tail mechanism, which can increase the availability of financial service for the relatively poor and even vulnerable groups, enhance the "hematopoietic" function for local economy and industry, and alleviate relative poverty in rural. With the continuing of “COVID-19” epidemic, Russia-Ukraine conflict, western inflation, crisis of energy, food and extreme climate, the global poverty rebounded and the gap between rich and poor is widening, in this background it has typical significance to investigate the mechanism and effect of mitigation of rural relative poverty by the inclusive digital finance in China. By the end of 2020, China lifted 98.99 million rural poor people out of poverty under the current standard as scheduled. After the eradication of absolute poverty, the governance of poverty will turn to consolidate and expand the achievements of poverty alleviation, and its core is to alleviate relative poverty and promote shared prosperity. So, what is the principle and mechanism of digital inclusive finance alleviating relative poverty? How effective is it to alleviate the relative poverty in rural households in China? These are the main questions to be studied in this paper. The structure of the paper is as follows. First, relevant theoretical literature is reviewed, and methods are introduced. Second, the principle and mechanism of how digital finance can alleviate relative poverty are analyzed by introducing a simple production function and Ramsey-Class-Koopmans model. Third, based on the latest data of China Household Finance Survey in 2019(CHFS2019) sponsored by Southwestern University of Finance and Economics, a mechanistic testing was performed, and finally, on the basis of this research, conclusions and suggestions are put forward.

## 2 Literature review

The poverty alleviation effect of digital finance has attracted wide attention from scholars at home and abroad in the past two years. First of all, most of the current literature believes that digital finance has shown a positive poverty mitigation effect due to its universality, so a large number of literature has studied the importance of digital finance alleviating poverty. Coleman (2007) pointed out that microfinance, as an important tool to fight against global poverty, will be increasingly established on the Internet platform in the future [1]. The World Bank indicates that fintech innovation holds promise for promoting financial inclusion and noted in its *World Development Report 2016*: *Digital Dividend* that digital technology and Internet development provide development opportunities for the poor and vulnerable through inclusion, efficiency and innovation [2]. Eugene O.I and Napoleon D.O (2017) found that digital financial services and products and universal financial knowledge popularization can effectively alleviate poverty in Nigeria [3]. Anju patwardhan (2018) believes that based on the innovation of products, business models and emerging technologies, fintech companies have promoted the development of financial inclusion and enriched the means of poverty alleviation [4]. Sun Jiguo, Han Kaiyan and Hu Jinyan (2022), based on CHFS2017 data of 153 cities above the prefecture level, found that digital finance can significantly alleviate relative poverty, but there are regional differences and urban-rural differences in poverty reduction effects [5]. Wang Gangzhen and Chen Mengjie (2020) demonstrated that digital inclusive finance in China has significant effect on slowing down absolute poverty and has spatial spillover effect [6]. There are also a few scholars who hold the opposite view, believing that digital finance has no poverty reduction effect and will even aggravate poverty. For example, the study of Wang Xiuhua and Zhao Yaxiong (2020) revealed that the impact of digital finance has Matthew effect, and the impact on poor households is not significant [7]. He Zongyue, Zhang Xun and Wan Guanghua (2020) believe that digital finance promotes the probability of poverty and the degree of multi-dimensional poverty, confirming the existence of the digital divide phenomenon [8].

Secondly, some literature has investigated the poverty mitigation mechanism of digital finance. They mainly focused on the financial equal empowerment mechanism, income growth and consumption promotion mechanism, and the mechanism of narrowing the gap between urban and rural areas. Banerjee and Newman (1993) conducted a qualitative analysis and concluded that the imperfect financial market access system is the main reason for income inequality and poverty, while the ubiquitous performance of digital finance overcomes the time-space limit and stimulates the credit mechanism for low-income groups [9]. Zhang Xun and Wan Guanghua (2019) studied the relationship between the development of digital economy, inclusive finance and inclusive growth, and found that China’s digital finance not only developed rapidly, but also significantly increased family income [10]. The calculation of Song Xiaoling (2017) and Xu Jiahui (2022) showed that the development of digital finance can reduce the relative poverty by significantly narrowing the income gap between urban and rural residents [11]. Ji Ming and Zeng Xihao (2022) discussed the promoting role of digital inclusive finance on poverty alleviation from the perspective of driving consumption [12]. In addition, there are a few scholars focusing on the long tail effect of digital financial problems. For example, Cui Xiaoxia (2018) analyzed the traditional financial “Two-eight rule”, and pointed out the highlight of digital financial is that it can include people with relatively low income as its service objects, and those people account for 80% of the total service group [13]. Xie Shengfeng (2021) measured the long tail effect of digital finance under the constraint of digital gap based on survey data of Hubei province [14].

The above literature lacks in-depth mathematical derivation when discussing the mechanism of digital finance alleviating relative poverty. In addition, the digital financial inclusion index provided by the Digital Finance Research Center of Peking University is simply applied in the above literature without the consideration of the heterogeneity of digital financial development in individual households. Besides, since Chris Anderson (2004) first put forward the Long Tail theory [15], few literature used this theory for the research of the financial field, and only a few domestic scholars made qualitative analysis. Therefore, the contributions of this paper are to break through the limitations of the long tail theory research focusing only on intelligence, media, consumption and other industries, and to conduct a mathematical derivation and quantitative research on the long tail effect of rural digital finance. After deriving the principle of digital finance alleviating relative poverty, we incorporate digital finance into a two-stage household consumption model, and deduce the long-tail mechanism for digital finance alleviating the relative poverty of farmers: productive investment mechanism, credit mechanism, financial asset allocation and entrepreneurial mechanism. Then, based on the CHFS2019 data, a digital financial measurement index for individual households is specially designed to verify the above mechanism. And according to practical problems, the paper proposes measures to improve the long-tail mechanism of rural digital financial development so as to alleviate relative poverty.

## 3 Methodology of the research

Firstly, the production function and utility function method are mainly included in the theoretical mechanism section. Referring to Barro(1990), finance variables is included in the traditional Cobb-Douglas production function [16], at the same time, since the fourth plenary session of the 19th National Congress of the Communist Party of China added "data" as a factor of production for the first time, which reflects the multiplier effect of the digital transformation in economic activities on improving production efficiency, Therefore, the digital technology of the labor force (represented by the Internet, mobile payment technology and other financial technology means) is included as the endogenous variable into the production function. Considering the influence of digital gap and assuming that the return of scale will remain unchanged, we analyzed the way digital finance affecting the changes of relative poverty in different stages under the constraints of the digital gap. Then we introduce Ramsey-Cass-Koopmans model and analyze the mechanism of increasing the overall utility of relative poor families, which alleviates their relative poverty, based on the two-stage household consumption and investment decision model with two phases in a life cycle, from the perspective of increasing productive investment opportunities and financial investment opportunities based on the utility function.

Secondly, the entropy synthesis method is used in the data processing. Since indicators such as the digital financial level, the relative poverty level and the digital ability of Chinese rural households are all multi-dimensional, while the entropy method is suitable for the synthesis, because the index weight is determined according to the variation degree of the index value, so it can avoid the bias caused by subjective factors. Finally, as a classical method for mechanism test, mediation effect test method is introduced. We carry out the causal stepwise regression test or called "three-step" test as it is widely used because it is easy to understand and interpret.

## 4 Theory and mechanism of digital finance alleviating relative poverty of rural households

The theoretical derivation is divided into two parts. First the principle of digital finance alleviating relative poverty is derived through a simple improved Cobb-Douglas production function method, and then the Ramsey-Class-Koopmans model is applied to analyze the mechanism how digital finance alleviates the relative poverty of rural families.

### 4.1 Theoretical derivation of digital finance alleviating rural relative poverty

Consider a closed small economy, including a developed sector with a small number of relatively rich groups and a backward sector with a large number of relatively poor groups, and the representative manufacturers of the two sectors satisfy the traditional Cobb-Douglas production function:

$$\text{Y}={\text{AK}}_{}^{\alpha }{\text{L}}_{}^{\beta }$$ (1)

In the formula, Y, A, K and L represent output (income), exogenous social production technology, capital (land) and labor respectively, and α and β are the production elasticity of capital (land) and labor respectively. It is assumed that this function has the characteristics of constant scale income. In the light of the production function, the digital technology (including internet, mobile payment technology and other fintech means) of labor force is included in the production function. The ef and gef respectively represent the development degree of digital finance of the two sectors. From this simple model, we can see that the larger the g value is, the smaller the gap between the two sectors will be, that is, the more developed the digital inclusive finance is, the greater the long tail effect will be. When g equals 1, it means digital finance reaches the highest inclusive stage, and the long tail effect reaches the maximum value. In the following, formulas with inferior 1 and 2 represent the economic variables of relatively developed sectors and relatively backward sectors respectively, and the formula with no inferior represents the total variables of the economy. For the relatively developed sector manufacturers, their representative production function is:

$${\text{Y}}_{\text{1}}={\text{A}}_{\text{1}}{\text{K}}_{1}^{\alpha_{1}}{({\text{efL}}_{\text{1}}\text{)}}^{\beta_{1}}$$ (2)

Similarly, the production function of the representative manufacturers in the relatively backward departments is as follows:

$${\text{Y}}_{\text{2}}={\text{A}}_{\text{2}}{\text{K}}_{2}^{\alpha_{2}}{\text{(}g\text{ef}L_{\text{2}}\text{)}}^{\beta_{2}}$$ (3)

We assume the relative poverty index of the two sectors is RP (Relative Poverty), since the main factor affecting the relative poverty is the income difference between sectors. RP is simplified as the ratio of the wage w of the two sectors, that is, RP = w_1_ / w_2_, the smaller the RP is, the lower the relative poverty level will be. In the case of steady-state equilibrium, each production factor is paid according to the marginal product, *w* = ∂*Y*/∂*L*. The incomes of the two sectors are as follows:

$$w_{1}=\frac{\partial Y_{1}}{\partial L_{1}}=\beta_{1}{\left(ef\right)}^{\beta_{1}}A_{1}K_{1}^{\alpha_{1}}L_{1}^{\beta_{1}-1}$$ (4)

$$w_{2}=\frac{\partial Y_{2}}{\partial L_{2}}=\beta_{2}{\left(gef\right)}^{\beta_{2}}A_{2}K^{\alpha_{2}}L_{2}^{\beta_{2}-1}$$ (5)

The relative poverty index is:

$$RP=\frac{w_{1}}{w_{2}}=\frac{\beta_{1}{\left(ef\right)}^{\beta_{1}}A_{1}K_{1}^{\alpha_{1}}L_{1}^{\beta_{1}-1}}{\beta_{2}{\left(gef\right)}^{\beta_{2}}A_{2}K_{2}^{\alpha_{2}}L_{2}^{\beta_{2}-1}}$$ (6)

Then we obtain the derivative of RP to digital finance ef:

$$\frac{\partial RP}{\partial ef}=(\beta_{1}-\beta_{2})\frac{{\left(ef\right)}^{\beta_{1}-\beta_{2}-1}\beta_{1}A_{1}K^{\alpha_{1}}L_{1}^{\beta_{1}-1}}{g^{\beta_{2}}\beta_{2}A_{2}K_{2}^{\alpha_{2}}L_{2}^{\beta_{2}-1}}$$ (7)

The production scale is assumed to be constant, so the formula is β_1_-β_2_ = (1-α_1_)-(1-α_2_) = α_2_-α_1_. In the early stage of economic development, especially in the era of agricultural economy, the backward sector is the agricultural sector, and its main capital is land, which has higher output elasticity than non-agricultural sector’ capital, namely α_2_-α_1_>0,β_1_-β_2_>0, ∂*RP*/∂*ef*>0. It shows that in the early stage of digital inclusive finance development, the backward sector has lower flexibility of labor output as the labor technology and information receiving capacity are weaker than the developed sector, which deepens the relative poverty. In this stage, the greater the digital gap is or the smaller the g value is, the greater the relative poverty will be. When β_1_ = β_2_, ∂*RP*/∂*ef* = 0, the digital gap and relative poverty reach their peak. In the advanced stage of economic development, with the rapid development of the tertiary industry such as financial technology and information, the output elasticity of the capital in the developed sectors is bigger than that of the backward sectors (as the land is its main capital), namely α_2_-α_1_>0, β_1_-β_2_>0, ∂*RP*/∂*ef*>0. It shows that with the development of information technology in the advanced stage, the development of fintech plays a role in inhibiting relative poverty. As the digital gap gradually declines, the relative poverty level will decline at a higher rate.

### 4.2 Theoretical derivation of the long tail mechanism

The following is the analysis of the mechanism by which relatively poor households who have just got rid of absolute poverty increase their overall consumption utility then alleviate relative poverty through productive investment opportunities and financial investment opportunities in the context of digital finance. Here, productive investment opportunities refer to the existence of a certain production technology (represented by the aforementioned production function) in the economy to realize inter-temporal conversion of consumer goods. Financial investment opportunities refer to the existence of loan opportunities in the economy constrained by loan vouchers. There are not only loan certificates, but also bonds, stocks and other capital market securities in modern society. Therefore, financial investment opportunities should also include consumers’ allocation behavior of financial assets and financial asset investment.

According to the Ramsey-Cass-Koopmans model, considering a two-stage (t, t+1) family consumption model with two periods in a life cycle, there are two types of households, relatively poor families X with a larger quantity and relatively wealthy families Y with a smaller quantity. The X and Y families can be regarded as the representative groups of the backward sector and the developed sector in the above theoretical analysis. The initial natural endowments of the two stages are c_t_ = x_t_, c_t+1_ = x_t+1_ for family X, and c_t_ = y_t_, c_t+1_ = y_t+1_ for family Y, where x_t_<y_t_, x_t+1_<y_t+1_.

We will focus on analyzing the consumption-investment decision-making behavior of the relatively poor rural X. In the context of the development of digital finance, since both digital technology and finance are endogenously incorporated into the production function and capital market loan is also driven by digital technology, both the consumer’s productive investment opportunities and financial investment opportunities are stimulated. Let the equilibrium interest rate of the capital market be r when the loan market clears. Its mathematical function is as follows:

$$\underset{c_{t},i_{t}^{X},c_{t+1}}{\text{max}}u^{X}(c_{t},c_{t+1})$$ (8)

$$s.t.0\leq c_{t}\leq x_{t},0\leq c_{t+1}\leq x_{t+1}+f^{X}(i_{t}^{X},A_{t}(T,ge)+(1+r)(x_{t}-i_{t}^{X}-c_{t})$$ (9)

$i_{t}^{X}$ is the investment of X in period t (expressed as the quantity of consumer goods invested), which means that consumer goods also have productive uses and can be converted into productive investment goods. It has nothing to do with *u*^*X*^ and the natural consumption endowment, but only is associated with the production function *f*^*X*^ of X and the equilibrium interest rate level r. Here, the production function also includes the technology At, which includes the general production technology status T, and the financial technology e which affects the development of digital finance, and the popularity of financial technology g which mainly measures the information gap reflecting the gap between family X and family Y in terms of information and technology and fintech. Fintech e can overcome the diminishing scale effect in the traditional production function, and has a long tail effect, that is, as the number of targets of financial factors increases, the marginal cost increases very little, or there will even be zero marginal cost. Therefore, a large number of relatively poor households like X can be included in the target of financial factors.

The two-stage consumption of the family satisfies:

$$c_{t}+\frac{1}{1+r}\cdot c_{t+1}=A_{t}W_{t}$$ (10)

W_t_ is the initial asset or income of the family. Since the X family is a relatively poor family, the W value is lower than a certain proportion of the average value of social family assets or income. To solve the optimization problem of Eq (8), let the household utility function satisfy the common constant relative risk aversion utility function (CRRA):

$$u(c_{t})=\frac{c_{t}^{1-\theta }-1}{1-\theta }\theta >0,\theta \neq 1$$ (11)

Its relative risk aversion coefficient θ is constant. On the optimal consumption path, according to the Euler equation, the relative relationship between the two periods of consumption can be deduced as:

$$\frac{c_{t+1}}{c_{t}}={\left(\frac{1}{\beta (1+r)}\right)}^{-\frac{1}{\theta }}$$ (12)

In formula (12), ß represents time preference, and ß>-1. In period t, consumers divide their natural endowment or labor income w into consumption and savings, and save the savings to the next period as the capital stock for the next period. Substituting Eq (12) into Eq (10), the ratio s of savings to income can be obtained. Assuming that the capital market is complete, the savings are all converted into the next investment through the capital market loan, so the ratio of savings to income can also be regarded as the loan ratio L(r), and is expressed as:

$$L(r)=s=\frac{{\left(1+r\right)}^{(1-\theta )/\theta }}{{\left(1+\beta \right)}^{1/\theta }+{\left(1+r\right)}^{(1-\theta )/\theta }}$$ (13)

$$\frac{d}{dr}[{\left(1+r\right)}^{(1-\theta )/\theta }]=[(1-\theta )/\theta ]{\left(1+r\right)}^{(1-2\theta )/\theta }$$ (14)

According to formula (13), if θ>1, L(r) is a decreasing function of r; if θ<1, L(r) is an increasing function of r. The general literature sets θ to be 1–4, so in general, L(r) is a decreasing function of r. As the cost of digital financial loans decreases, household savings and the proportion of loans will increase, which will increase the investment in the next period through loans, and then increase the overall consumption utility. For the relatively poor, the relative risk aversion coefficient θ is larger than that of the relatively rich, that is, the marginal utility of consumption is more elastic, so the marginal borrowing of the relatively poor increases more, which will not only improve its overall utility, but also improve social welfare. It needs to be clearly pointed out that the essence of digital finance reducing borrowing costs is that digital technology reduces the degree of information asymmetry between the relatively poor and financial capital suppliers. At the same time, the network search cost and the network zero marginal cost effect greatly reduce the threshold for financial capital providers to provide financial services to the relatively poor, so that the accessibility of financial services to the relatively poor is greatly improved.

In the above model, how does household X make consumption and investment decisions? In fact, according to the optimization conditions, it can be seen that the productive investment $i_{t}^{X}$ of X can be summarized as follows:

$$\underset{i_{t}^{X}}{\text{max}}(x_{t}-i_{t}^{X}+\frac{x_{t+1}+f^{X}(i_{t}^{X},A_{t}(T,ge))}{1+r})$$

$$\text{i}.\text{e}.\;\underset{i_{t}^{X}}{\text{max}}(\frac{f^{X}(i_{t}^{X},A_{t}(T,ge))}{1+r}-i_{t}^{X})$$ (15)

After solving the above rule problem to obtain the appropriate investment level, the consumption decision of X is equivalent to Eqs (8) and (9). This also means that Fisher’s Separation Theorem is established, that is, X’s consumption decision and investment decision are separated, and the investment decision will be given priority, based on which the consumption decision will be considered. Digital finance will expand the utility and welfare of the relatively poor from both production investment and financial investment. First, the emergence of financial technology will improve the production technology, namely the digital technology of the labor force (represented by the Internet, mobile payment technology, etc.) that as an exogenous variable can be incorporated into the traditional production function, which acts to expand output, and makes $\partial f^{X}/\partial i_{t}^{X}\rangle 0$. According to the knowledge of microeconomics, the production conversion curve is used to indirectly to represent the production function. Second, through the introduction of capital market lending and financial asset investment, the utility of the relatively poor is further enhanced.

The mathematical programming form of X’s consumption-investment decision problem is shown in1, which is reconstructed by the author referring to the book *Financial Economics* [17].

**Fig 1:**
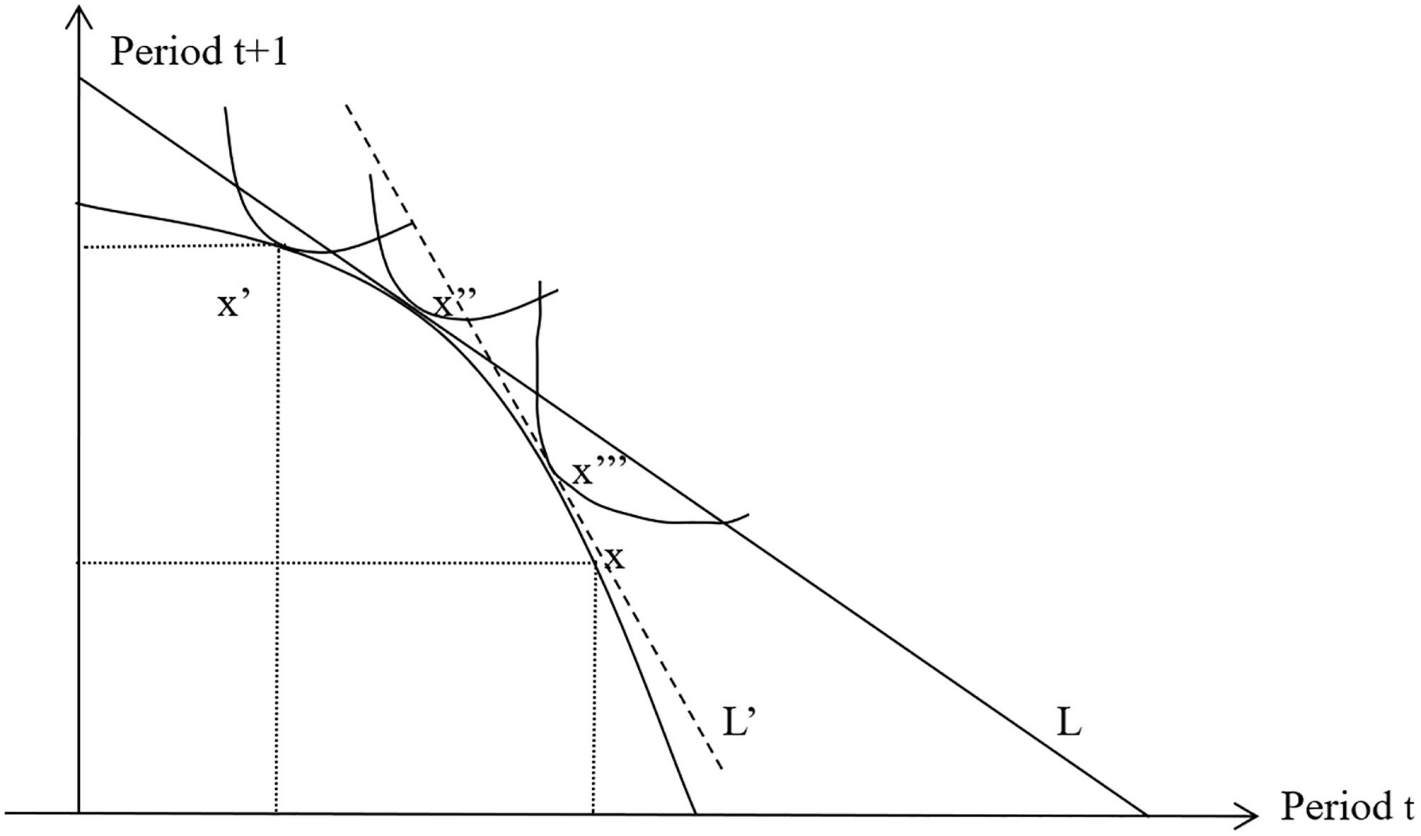
Digital finance enhancing the utility of relatively poor household X by promoting productive and financial investment. Note: Reconstructed by the author.

Fig 1 shows that X moves the consumption endowment from the natural endowment x to x’ by the productive investment i_t_, and then move it from x’ to x’’ through borrowing in the financial market. Therefore, X expands the set of consumption choices through productive investment, and then further expands it through financial investment (here as negative investment), thus causing the overall right shift of the utility function, thereby achieving an improvement in its overall consumption utility level. And digital financial inclusion further reduces the cost of borrowing and increases the slope $\frac{1}{1+r}$ of the loan curve L, and finally make the consumer indifference curve, the new loan curve L’ and the production conversion curve tangent to x‴, thus increasing the final consumption endowment from point x’’ to point x‴, thus causing the overall right shift of the consumer indifference curve, thereby achieving an improvement in its overall consumption utility level. We can also analyze the consumption and investment decisions of relatively wealthy household Y. The difference is that in the process of the development of digital inclusive finance for relatively poor family X, as the information gap g decreases, its marginal utility relative to family Y will increase faster, which promotes the improvement of shared prosperity.

From the above mathematical analysis, it can be concluded that digital finance alleviates relative poverty mainly by increasing the productive investment opportunities and financial investment opportunities for relatively poor families, which is embodied in the credit mechanism with a precise increase of credit availability for the relatively poor, the inter-temporal allocation mechanism to promote the transformation of savings and investment, the fixed asset investment mechanism and financial asset allocation mechanism to increase productive investment opportunities, and the entrepreneurial mechanism to increase income. The above mechanisms can alleviate the income, opportunity and right inequality of the relatively poor, so as to promote the realization of shared prosperity.

## 5 Empirical study of the long tail effect of digital finance alleviating relative poverty

### 5.1 Hypothesis and methods

Based on the above derivation and mechanism analysis, we propose four research hypotheses. Research hypothesis 1: digital finance alleviates relative poverty by increasing productive investment opportunities; research hypothesis 2: digital financial inclusion alleviates relative poverty by increasing household credit availability; research hypothesis 3: digital finance enhances the wealth of the relatively poor by enhancing the allocation of financial assets; research hypothesis 4: digital finance alleviates relative poverty by promoting entrepreneurship of rural households.

In order to verify the above-mentioned internal mechanisms of digital finance alleviating relative poverty, this paper uses the mediation effect model to analyze the indirect effect of digital finance on relative poverty alleviation. The following mediation effect model is established:

$$\text{RP}=\alpha_{1}+\beta_{1}\text{DF}+\gamma_{1}$$ (16)

$$\text{M}=\alpha_{2}+\beta_{2}\text{DF}+\gamma_{2}$$ (17)

$$\text{RP}=\alpha_{3}+\beta_{3}\text{M}+\gamma_{3}$$ (18)

$$\text{RP}=\alpha_{4}+\beta_{4}\text{DF}+\beta_{5}\text{M}+\gamma_{4}$$ (19)

Among them, M is the mediator variable, and this paper includes the productive investment (M_1_), household formal credit (M_2_), financial asset allocation (M_3_, mainly stocks, bonds, and wealth management products) and rural household entrepreneurship (M_4_). The explanatory variable is the digital financial index DF and the explained variable is the multidimensional relative poverty RP. The effect of DF on RP through M is the mediating effect. In the above model, the corresponding total effect is β_1_, β_2_ is the effect of the independent variable DF (digital finance) on the intermediary variable M, and β_3_ is the effect of the intermediary variable M on the dependent variable RP after controlling the influence of the independent variable DF (digital finance). β_4_ and β_5_ are the effects of both independent variables and mediating variables on the dependent variable.

### 5.2 Variable design, data source and sample descriptive statistics

The explained variables, explanatory variables, mediating variables and control variables, as well as the description of the processing methods in data extraction in this empirical study, are shown in Table 1.

**Table 1:** Variable definitions and measurement.

Variable	Symbol	Variable Item	Variable Definition and Measurement
Explained Variable	RP	Household Relative Poverty Index	Synthesis based on multi-dimensional indicators using entropy weight method
Explanatory Variable	DF	Digital Financial Level	Synthesis of multiple indicators according to the entropy weight method
Mediating Variable	M_1_	Productive Investment;	If there is productive fixed asset investment, assign a value of 1; if not, assign a value of 0.
	M_2_	Household Formal Credit;	If there is bank credit, assign 1; if not, assign 0.
	M_3_	Financial Asset Allocation;	If there are financial assets, assign a value of 1; if not, assign a value of 0.
	M_4_	Household Entrepreneurship	Number of startups
Control Variable	C_1_	Household Size	Number of household members
	C_2_	Marital Status	Unmarried, assign 0; married, assign 1
	C_3_	Household Savings	Less than 0, assign 0; [0, median 2000 yuan], assign 1; greater than 2000 yuan, assign 2.
	C_4_	Information Ability	Synthesis according to entropy weight method.
	C_5_	Party membership	Yes, assign 1; No, assign 0.
	C_6_	Family members in employment	Yes, assign 1; No, assign 0.

Among them, the family common wealth index RP is determined by multi-dimensional factors such as income, education level, medical health, living standard, social interaction, and subjective well-being. The construction of multi-dimensional indicators is shown in Table 2. Finally, the entropy value method is used to synthesize the RP value of each family.

**Table 2:** Construction of multidimensional relative poverty indicators for households.

Dimension	Index	Relative Poverty Threshold and Description
Income	Household Incomes Per Capita	With reference to the National Bureau of Statistics, the per capita disposable income of households in 2019 is classified into low, medium and high groups. Rural households are assigned a value of 2 if their incomes are less than 4,263 yuan, 1 if they are between 4,263 and 13,984 (inclusive), and 0 if they are greater than 13,984. Urban households are assigned a value of 2 if their incomes are less than 15,549 yuan, 1 if between 15,549 and 37,876 (inclusive), and 0 if greater than 37,876.
Educate	Years of Education	Family members aged 16 and above with less than 9 years of education are assigned a value of 1, otherwise, a value of 0.
Healthy	Medical Insurance	As long as one of the family members does not have medical insurance, the value is 1, otherwise the value is 0.
	Physical Conditions	If the value of the physical condition is greater than 3, the value is assigned as 1, and the rest is assigned as 0 (the health survey options are: 1. Very good; 2. Good; 3. Average; 4. Poor; 5. Very poor).
Standard of Living	Houses	If there is no self-owned house, it is assigned a value of 1, and the rest is assigned a value of 0.
	Vehicles	If there is no private car, it is assigned a value of 1, and the rest are assigned a value of 0.
Subjective Well-being	Happiness	If the happiness value is greater than 3, the value is assigned as 1, and the rest is assigned as 0 (the survey options are: 1. very happy; 2. happy; 3. average; 4. Unhappy; 5. Very unhappy).
Social Interaction	Social Interaction	Yes, assign 0; No, assign 1.

Secondly, the county-level digital financial inclusion index compiled by the Digital Finance Research Center of Peking University and the self-designed household digital financial index are included as explanatory variables. Most of the existing literature uses the former to directly measure the digital finance level of each sample household. This study believes that the advantage of this indicator is that it comprehensively reflects the coverage breadth, depth and digitalization degree, while when describing the digital finance development level of specific households, there are deficiencies, because the index more reflects the overall development level of digital financial inclusion in the city or county where it is located, and cannot accurately reflect the situation of a specific sample of rural households. Therefore, in order to more accurately describe the level of digital finance at the household level, seven indicators of economic behavior of family members engaged in non-cash transactions through the Internet and other channels are specially selected, as shown in Table 3, and then the digital financial index is synthesized by the entropy weight method. The county-level digital financial inclusion index compiled by the Digital Finance Research Center of Peking University is used as a substitute explanatory variable to participate in the robustness test.

**Table 3:** Composition indicators of digital finance index.

Indicator	Description
Online Shopping Expenses	If there is online shopping cost, the value is 1; if there is no network cost, the value is 0.
Third-party Account	If opened, assign 1; if not, assign 0.
Internet Financial Management Confirmation	If yes, assign 1; if not, assign 0.
Internet Loan	If yes, assign 1; if not, assign 0.
QR Code Collection Frequency	If the QR code is used, assign the value 1; if not, assign the value 0.
Proportion of Revenue from QR Code Receipts	If yes, assign 1; if not, assign 0.
Confirmation of Credit Card Use	If it is used, assign 1; if not, assign 0.

The information capability index of the control variables is synthesized by the entropy method from the variables including household communication expenses, possession of computers and mobile phones, holding quantity of TVs, Internet access fees and the number of illiterate family members.

The data of farmer households in this empirical study is from the CHFS database in 2019. The total sample size of the database is 34,643 households, including 22,307 urban households and 12,336 rural households. After removing some samples which miss values, the final sample includes 20,231 urban households and 11,519 rural households. The descriptive statistics of each variable of rural households are shown in Table 4.

**Table 4:** Descriptive statistics of variables.

VARIABLES	N	mean	sd	min	max
RP	11,519	0.1420	0.1310	0	1
DF	11,519	0.0517	0.0942	0	0.8410
M_1_	11,519	0.0073	0.0851	0	1
M_2_	11,519	0.0069	0.0831	0	1
M_3_	11,519	0.0107	0.1030	0	1
M_4_	11,519	0.1810	0.6120	0	20
C1	11,519	3.0710	2.1120	0	20
C2	11,519	0.8620	0.3450	0	1
C3	11,519	0.8370	0.9260	0	2
C4	11,519	0.1220	0.3270	0	1
C5	11,519	0.7990	0.4000	0	1
C6	11,519	0.3230	0.3380	0	1

### 5.3 Analysis of empirical results

#### 5.3.1 Test of mediation effect

(1) Digital finance, productive investment and relative poverty

In Table 5 of the empirical results, column 2 shows that digital finance can significantly reduce relative poverty at the 1% level, and the significance coefficient reaches -0.121, and all control variables also significantly reduce relative poverty, that is, the relative poverty degree is lowered greatly in households of a large household size, a married head of household, high household savings, a strong information capacity, party membership, and household members in employment. Column 3 indicates that digital finance can significantly promote productive investment at the 1% level, and family size and savings also have a significant effect. Column 4 illustrates that productive investment can significantly reduce relative poverty at the 10% level. Column 5 describes that after adding productive investment variables, the absolute value of the influence coefficient of -0.122 is greater than the regression result of column 2, which is -0.121. That proves the intermediary variable of productive investment does play a role in reducing relative poverty in digital finance. Based on the above analysis, the mediation effect is established, that is, digital finance alleviates the poverty of the relatively poor by increasing productive investment opportunities, because the development of digital finance brings financial services to households, reduces the difficulty of obtaining productive investment, and promotes the production, thereby alleviating the poverty of the relatively poor. So hypothesis 1 holds.

**Table 5:** Test of the mediating effect of productive investment.

VARIABLES	RP	M1	RP	RP
X	-0.121***	0.220***	-	-0.122***
	(0.0134)	(0.0092)	-	(0.0138)
C_1_	-0.0022***	0.0008**	-0.0027***	-0.0022***
	(0.0005)	(0.0004)	(0.0005)	(0.0005)
C_2_	-0.0459***	0.0003	-0.0460***	-0.0459***
	(0.0034)	(0.0023)	(0.0034)	(0.0034)
C_3_	-0.0257***	-0.0023***	-0.0276***	-0.0257***
	(0.0013)	(0.0009)	(0.0013)	(0.0013)
C_4_	-0.0168***	0.0015	-0.0171***	-0.0168***
	(0.0035)	(0.0024)	(0.0035)	(0.0035)
C_5_	-0.0500***	0.0003	-0.0514***	-0.0500***
	(0.0029)	(0.0019)	(0.0029)	(0.0029)
C_6_	-0.0421***	-0.0020	-0.0536***	-0.0421***
	(0.0037)	(0.0026)	(0.0035)	(0.0037)
M_1_	-	-	-0.0244*	0.0019
	-	-	(0.0133)	(0.0136)
Constant	0.272***	-0.0046*	0.274***	0.272***
	(0.0037)	(0.0026)	(0.0037)	(0.0037)
Observations	11,519	11,519	11,519	11,519
R-squared	0.155	0.058	0.149	0.155

Note: The fourth column is the regression of the mediator variable on the dependent variable, and the fifth column is the regression of the independent variable and the mediator variable on the dependent variable, the same below. Significance levels are set at 0.01, 0.05 and 0.1.

(2) Digital finance, household formal credit and relative poverty

Column 2 in Table 6 shows that digital finance can significantly reduce relative poverty at the 1% level, with a coefficient of -0.121, and all control variables also significantly reduce relative poverty, that is, the relative poverty degree is lowered greatly in households of a large household size, a married head of household, high household savings, a strong information capacity, party membership, and household members in employment. Column 3 demonstrates that digital finance can significantly promote household formal credit at the 1% level, and household size also significantly promotes household credit, because the more members the household has, the stronger their risk tolerance is, and the stronger their propensity to borrow is. Column 4 notes that household credit can significantly reduce relative poverty at the 10% level. Column 5 indicates that after considering the impact of household credit, digital finance can still significantly reduce relative poverty at the 1% level. Based on the above analysis, the mediation effect is established, that is, digital finance will alleviate the poverty of the relatively poor by increasing household credit. This is because the development of digital finance has reduced the cost of obtaining credit for households, sent loans to households faster and more conveniently, and increased the financial strength of household development, thereby alleviating the poverty of the relatively poor. So hypothesis 2 holds.

**Table 6:** Mediating effect test of household formal credit.

VARIABLES	RP	M_2_	RP	RP
DF	-0.121***	0.197***	-	-0.122***
	(0.0134)	(0.0090)	-	(0.0137)
C_1_	-0.0022***	0.0012***	-0.0027***	-0.0022***
	(0.0005)	(0.0004)	(0.0005)	(0.0005)
C_2_	-0.0459***	0.0009	-0.0460***	-0.0459***
	(0.0034)	(0.0023)	(0.0034)	(0.0034)
C_3_	-0.0257***	-0.0019**	-0.0276***	-0.0257***
	(0.0013)	(0.0009)	(0.0013)	(0.0013)
C_4_	-0.0168***	0.0005	-0.0171***	-0.0168***
	(0.0035)	(0.0023)	(0.0035)	(0.0035)
C_5_	-0.0500***	0.0003	-0.0515***	-0.0500***
	(0.0029)	(0.0019)	(0.0029)	(0.0029)
C_6_	-0.0421***	-0.0032	-0.0537***	-0.0421***
	(0.0037)	(0.0025)	(0.0035)	(0.0037)
M_2_	-	-	-0.0209*	0.0037
	-	-	(0.0137)	(0.0139)
Constant	0.272***	-0.0053**	0.274***	0.272***
	(0.0037)	(0.0025)	(0.0037)	(0.0037)
Observations	11,519	11,519	11,519	11,519
R-squared	0.155	0.049	0.149	0.155

(3) Digital finance, financial asset allocation and relative poverty

Column 2 in Table 7 shows that digital finance can significantly reduce relative poverty at the 1% level, with a significance coefficient of -0.121, and all control variables also significantly reduce relative poverty as disclosed before. Column 3 demonstrates that digital finance can significantly promote the rational allocation of financial assets at the level of 1%. Among them, more household savings and more working members reflect the stability of the family, and can also significantly facilitate the allocation of financial assets. Column 4 notes that financial asset allocation can significantly reduce relative poverty at the 1% level. Column 5 indicates that while after adding financial asset allocation variables, the absolute value of the influence coefficient of -0.119 is smaller than the regression result of column 2, which is -0.121. That proves the mediator variable of financial asset allocation does play a role in the process of digital finance reducing relative poverty, and it can still significantly reduce relative poverty at the 1% level. Based on the above analysis, the mediation effect is established, that is, digital finance can alleviate the poverty of the relatively poor by strengthening the allocation of financial assets. This is because with the introduction of digital finance into thousands of households, the financial literacy of households has been improved, the awareness of rational allocation of financial assets has gradually increased, and the development of household financial behavior has alleviated the poverty of the relatively poor. Therefore, hypothesis 3 holds.

**Table 7:** Test of mediation effect of financial assets allocation.

VARIABLES	RP	M_3_	RP	RP
DF	-0.121***	0.131***	-	-0.119***
	(0.0134)	(0.0113)	-	(0.0135)
C_1_	-0.0022***	-0.0007	-0.0028***	-0.0023***
	(0.0005)	(0.000459)	(0.0005)	(0.0005)
C_2_	-0.0459***	0.00242	-0.0459***	-0.0458***
	(0.0034)	(0.00285)	(0.0034)	(0.0034)
C_3_	-0.0257***	0.00654***	-0.0274***	-0.0255***
	(0.0013)	(0.00107)	(0.0013)	(0.0013)
C_4_	-0.0168***	-0.00225	-0.0172***	-0.0169***
	(0.0035)	(0.00290)	(0.0035)	(0.0035)
C_5_	-0.0500***	-0.000180	-0.0515***	-0.0500***
	(0.0029)	(0.00242)	(0.0029)	(0.0029)
C_6_	-0.0421***	0.0189***	-0.0532***	-0.0417***
	(0.0037)	(0.0031)	(0.0035)	(0.0037)
M_3_	-	-	-0.0289***	-0.0184*
	-	-	(0.0111)	(0.0111)
Constant	0.272***	-0.0072**	0.274***	0.272***
	(0.0037)	(0.0031)	(0.0037)	(0.0037)
Observations	11,519	11,519	11,519	11,519
R-squared	0.155	0.033	0.150	0.155

(4) Digital finance, household entrepreneurship and relative poverty

Column 2 in Table 8 shows that digital finance can significantly reduce relative poverty at the 1% level, with a significance coefficient of -0.121, and all control variables also significantly reduce relative poverty, that is, the relative poverty degree is lowered greatly in households of a large household size, a married head of household, high household savings, a strong information capacity, party membership, and household members in employment. Column 3 demonstrates that digital finance can significantly promote family entrepreneurship at the 1% level, with a significant coefficient of 2.276. Among them, married families with more family savings and more working members have greater risk tolerance, and are affected by the development of digital finance. As a result, the number of households engaged in entrepreneurship has also increased significantly. Column 4 notes that family entrepreneurship can significantly reduce relative poverty at the 1% level. Column 5 indicates that after adding the family entrepreneurship variable, the absolute value of the influence coefficient -0.111 is instead smaller than the regression result -0.121 of column 2, which shows that the family entrepreneurship mediator variable does play a role in the impact of digital finance on alleviating relative poverty, and it can still significantly reduce relative poverty at the 1% level. Based on the above analysis, the mediation effect is established, that is, the development of digital finance can significantly increase household entrepreneurship, thereby alleviating the poverty of the relatively poor. This is because digital finance provides more channels for families to obtain funds, breaks the constraints of time and space, optimizes the efficiency of labor market allocation, provides flexible employment and self-employment opportunities, and can greatly increase family assets and reduce relative poverty to achieve shared prosperity. So hypothesis 4 holds.

**Table 8:** Mediating effect test of family entrepreneurship.

VARIABLES	RP	M_4_	RP	RP
DF	-0.121***	2.276***	-	-0.111***
	(0.0134)	(0.0629)	-	(0.0142)
C_1_	-0.0022***	-0.0021	-0.0027***	-0.0023***
	(0.0005)	(0.0026)	(0.0005)	(0.0005)
C_2_	-0.0459***	0.0380**	-0.0456***	-0.0457***
	(0.0034)	(0.0159)	(0.0034)	(0.0034)
C_3_	-0.0257***	0.0131**	-0.0272***	-0.0256***
	(0.0013)	(0.0059)	(0.0013)	(0.0013)
C_4_	-0.0168***	0.0083	-0.0170***	-0.0168***
	(0.0035)	(0.0162)	(0.0035)	(0.0035)
C_5_	-0.0500***	-0.0142	-0.0514***	-0.0501***
	(0.0029)	(0.0135)	(0.0029)	(0.0029)
C_6_	-0.0421***	0.106***	-0.0509***	-0.0416***
	(0.0037)	(0.0174)	(0.0035)	(0.0037)
M_4_	-	-	-0.0096***	-0.0046**
	-	-	(0.0019)	(0.0019)
Constant	0.272***	0.0019	0.274***	0.272***
	(0.0037)	(0.0175)	(0.0037)	(0.0037)
Observations	11,519	11,519	11,519	11,519
R-squared	0.155	0.148	0.151	0.155

#### 5.3.2 Endogeneity test and robustness test

First, the p-value of the model is greater than 0 through the Hausman test, and the null hypothesis is accepted, indicating that the explanatory variable X is an exogenous explanatory variable and has nothing to do with the random error linearly. Secondly, when doing the robustness test, the variable X is replaced with the digital financial inclusion index provided by the Digital Economy Research Center of Peking University, and it is found that only M_2_ and M_4_ passed the test, as shown in Tables 9 and 10. Table 9 shows that the digital financial inclusion index can significantly promote productive credit at the 5% level, with a coefficient of 0.0321; when X variable of digital financial inclusion is controlled and not controlled, credit has an impact on relative poverty at the level of 10% and 5%, and with coefficients of -0.192 and -0.185, respectively.

**Table 9:** Robustness test of the mediation effect of financial asset allocation.

VARIABLES	RP	M_2_	RP	RP
DF	-0.380***	0.0321**	-	-0.375**
	(0.147)	(0.0126)	-	(0.1470)
C_1_	-0.0242***	0.0020***	-0.0243***	-0.0239***
	(0.0044)	(0.0004)	(0.0044)	(0.0044)
C_2_	-0.281***	0.0012	-0.281***	-0.281***
	(0.0272)	(0.0023)	(0.0272)	(0.0272)
C_3_	-0.251***	0.0013	-0.250***	-0.251***
	(0.0101)	(0.0009)	(0.0101)	(0.0101)
C_4_	-0.559***	0.0164***	-0.555***	-0.556***
	(0.0283)	(0.0024)	(0.0284)	(0.0284)
C_5_	-0.129***	0.0009	-0.129***	-0.129***
	(0.0282)	(0.0024)	(0.0282)	(0.0282)
C_6_	-0.368***	0.0026	-0.368***	-0.367***
	(0.0231)	(0.0019)	(0.0231)	(0.0231)
M_2_			-0.192*	-0.185**
			(0.0810)	(0.0180)
Constant	0.4170	-0.160***	-1.380***	0.3920
	(0.6960)	(0.0593)	(0.0298)	(0.6960)
Observations	11,519	11,519	11,519	11,519
R-squared	0.162	0.010	0.162	0.163

**Table 10:** Robustness test of the mediation effect of family entrepreneurship.

VARIABLES	RP	M_4_	RP	RP
DF	-0.380***	0.0544*	-	-0.374**
	(0.147)	(0.0906)	-	(0.147)
C_1_	-0.0242***	0.0079***	-0.0237***	-0.0233***
	(0.0044)	(0.0027)	(0.0044)	(0.0044)
C_2_	-0.281***	0.0408**	-0.277***	-0.277***
	(0.0272)	(0.0168)	(0.0271)	(0.0271)
C_3_	-0.251***	0.0509***	-0.245***	-0.246***
	(0.0101)	(0.0062)	(0.0101)	(0.0101)
C_4_	-0.559***	0.332***	-0.523***	-0.525***
	(0.0283)	(0.0172)	(0.0287)	(0.0287)
C_5_	-0.129***	0.0134	-0.128***	-0.128***
	(0.0282)	(0.0171)	(0.0282)	(0.0282)
C_6_	-0.368***	0.0135	-0.367***	-0.366***
	(0.0231)	(0.0143)	(0.0230)	(0.0230)
M_4_	-	-	-0.107***	-0.107***
	-	-	(0.0155)	(0.0155)
Constant	0.4170	-0.2980	-1.383***	0.3810
	(0.6960)	(0.4280)	(0.0297)	(0.6950)
Observations	11,519	11,519	11,519	11,519
R-squared	0.162	0.052	0.165	0.166

Table 10 demonstrates that the digital financial inclusion index can significantly promote family entrepreneurship at the 10% level, with a coefficient of 0.0544; with and without the control of the digital financial inclusion X variable, family entrepreneurship has a significant impact on relative poverty at the 5% level. influence, with a coefficient of -0.107.

#### 5.3.3 Heterogeneity test

By combining rural and urban data to test for heterogeneity, it is found that both urban and rural digital finance levels can significantly alleviate relative poverty, with coefficients reaching -1.446 and -0.578, respectively, as shown in Table 11.

**Table 11:** Urban-rural heterogeneity test of the impact of digital finance on relative poverty.

	RP-rural	RP-urban
X	-1.446*** (-12.97)	-0.578*** (-27.82)
C_1_	-0.0183*** (-4.18)	0.0111*** (5.55)
C_2_	-0.279*** (-10.34)	-0.134*** (-14.47)
C_3_	-0.228*** (-22.33)	-0.102*** (-26.70)
C_4_	-0.129*** (-4.60)	-0.224*** (-25.40)
C_5_	-0.351*** (-15.31)	-0.0253*** (-3.47)
C_6_	-0.424*** (-14.15)	-0.816*** (-35.87)
N	11294	18129

Note: The t statistic is in brackets, and the significance levels are 1%, 5%, and 10%.

It can be seen that the development of digital finance has a more obvious effect on alleviating relative poverty in rural areas than in urban areas. This is mainly due to the effect of the long tail mechanism of digital finance. Financial services have gradually extended from "head" customers in urban areas to rural "tail" customers.

## 6 Conclusions and policy recommendations

Based on the improved Cobb-Douglas production function and Ramsey-Cass-Koopmans two-stage household consumption model, this paper deduces the long-tail effect and mechanism of digital finance alleviating rural relative poverty. On the basis of quantitatively characterizing the poverty level of 11,519 rural households from the CHFS2019 data, using the mediation effect model to verify the four hypotheses derived from the long-tail mechanism, the main conclusions are as follows: first, digital finance can significantly and robustly increase the availability of formal credit for households and promote family entrepreneurship to alleviate relative poverty. Relying on financial technology, digital finance expands the mode and scope of financial services, reduce the risk of information asymmetry and borrowing costs, thus can break through the space bottleneck, benefit farmers in remote and scattered rural areas and solve financing difficulties of rural entrepreneurs by promoting credit availability. Second, digital finance can also alleviate relative poverty by increasing farmers’ productive investment opportunities and strengthening financial asset allocation, but the conclusion is not stable, which shows that rural digital finance can partially exert the long-tail effect. On the one hand, there are deficiencies in the blood making mechanism. Due to the backward development of rural industries, the effect of digital finance on increasing farmers’ income is not significant. Even if there is no financial constraints, relatively poor farmers are also difficult to expand productive activities due to the constraints in material and human capital, labor skills and economic behavior. On the other hand, the lagging development of the rural financial market, especially the capital market, makes it impossible to optimize the allocation of rural household assets, as the channels for digital financial management, insurance and investment are limited. Second, the heterogeneity test also demonstrates that digital finance can significantly alleviate the relative poverty of urban and rural residents at the same time, and the effect on rural areas is more obvious, which is essentially the embodiment of the effect of the long tail mechanism, and also highlights the importance of the development of rural digital finance. Finally, the relative poverty alleviation effect of digital finance is also affected by household information ability, household size, marital status, private loan and savings. For households of a large household size, a married head of household, high household savings, a strong information capacity, party membership, and family members in employment, relative poverty can also be reduced significantly.

Therefore, in order to improve the level of rural digital finance and alleviate relative poverty, the priority is to give full play to the “blood-making” function under the long-tail mechanism of digital finance, through the effective docking of the digital finance supply chain and the whole industry chain including the key rural industries (crops and seedlings), emerging industry (such as the tourism industry) and green industry, to support the advanced rural industrial structure and green transformation development, innovate the "digital finance + rural organization (or enterprise) + farmers" model, and increase farmers’ productive investment opportunities and innovation and entrepreneurship ability, so as to cultivate new driving force of endogenous income growth and wealth. Secondly, it is necessary to actively develop customized digital wealth management, digital insurance and securitized products for rural groups and cultivate the rural capital market, to allow farmers to enjoy the benefits of wealth appreciation by optimizing the allocation of financial assets. In addition, great importance should be attached to the "Matthew effect" caused by the digital divide and digital poverty, strengthening the construction of rural digital infrastructure, improving supporting services related to digital technology, promoting the effective supply of information, enhancing the ability of farmers to use effective information, and narrowing the digital divide, to enable farmers to equally enjoy the digital dividend of the information age, break the “inclusive finance development paradox” and solve the problem of isolated information. Apart from that, it is important to establish and improve the risk prevention and supervision system of rural digital inclusive finance, strengthening rural data legislation and digital platform governance and at the same time preventing the phenomenon of "mission drift" and "elite capture" of financial institutions.

The limitation of this paper is that the classical mediation effect method for mechanism test is difficult to establish a comprehensive model including all the potential causal transmission routes. Due to the limitations of the survey data, the estimation of the indirect long tail effects may vary with different countries. The future direction is to perform international comparative studies so as to make policy proposals for the development of digital financial inclusion to meet the challenges in the task of eradicating global poverty.

## Supporting information

S1 Data (XLSX)
